# Micro-variations in timing and loudness affect music-evoked mental imagery

**DOI:** 10.1038/s41598-025-12604-4

**Published:** 2025-08-22

**Authors:** Ceren Ayyildiz, Andrew J. Milne, Muireann Irish, Steffen A. Herff

**Affiliations:** 1 Sydney Conservatorium of Music, The University of Sydney, Sydney, NSW Australia; 2 The MARCS Institute for Brain, Behaviour and Development, Western Sydney University, Sydney, NSW Australia; 3 Brain and Mind Centre, The University of Sydney, Sydney, NSW Australia; 4 School of Psychology, The University of Sydney, Sydney, NSW Australia

**Keywords:** Mental imagery, Music cognition, Individual differences, Micro-timing, Musical expression, Music-evoked mental imagery, Psychology, Human behaviour

## Abstract

Music can shape the vividness, sentiment, and content of directed mental imagery. Yet, the role of specific musical features in these effects remains elusive. One important aspect of human musical performances is the presence of micro-variations—small deviations in timbre, pitch, and timing, driven by motor and attentional processes. These variations enhance perceived “naturalness” compared to mechanical playing without such variations. Here, we investigated whether random micro-variation, as opposed to mechanical playing, affects mental imagery characteristics. One hundred participants performed a directed mental imagery task where they imagined a journey, accompanied either by drumming with micro-variation, drumming without micro-variation, or silence. Participants rated the vividness, distance and time travelled of their imagined content, alongside free-format content responses. Bayesian multilevel regression model showed that repetitive quasi-isochronous drumming enhanced mental imagery vividness, with a stronger effect observed when the drumming contained random micro-variation. Drumming with random micro-variation also increased imagined distance and time travelled compared with silence. Furthermore, individual traits in absorption, ability to imagine vividly, and level of musical training interacted with auditory conditions to further shape mental imagery characteristics. The findings have implications for the use of music to support imagery in creative, recreational, and therapeutic settings.

## Introduction

Music is an important component of human culture^1–4^. It considerably influences our mental and emotional states^5^ and can impact dimensions such as motivation^6^, decisions^7^, and general thoughts^8,9^. These thoughts can, for example, manifest as associations^10^, autobiographical memories^11^, daydreams^12^, and mind-wandering^13^. In many cases, music has the capacity to evoke mental imagery, where these thoughts are vividly imagined (i.e., perceived with clarity, detail, and intensity)^14–16^. Mental imagery allows individuals to simulate past and future scenarios^17,18^, adaptively interpret experiences^19,20^, and engage in effective planning^21^ and decision-making^20^. Despite mental imagery’s important part in human cognition, the mechanisms by which music influences it – particularly through musical features like timing and loudness – remain largely unknown^14,22^. Inspired by ethnomusicological accounts, we draw on the musical features involved in rituals traditionally designed to facilitate mental imagery^23–25^. Building on prior work into the role of micro-variation in music perception^26–28^, we explore the role of small, random variations in the musical signal on vividness, emotional sentiment, and spatiotemporal aspects of music-evoked mental imagery.

Mental imagery is defined as a mental simulation of a mono- or multi-modal experience in the absence of the corresponding physical stimulus, whilst being aware of the counterfactual nature of the experience^29–31^. In addition to its everyday role in cognition such as simulating future scenarios^32^, it is often used in recreational^33^ and clinical applications, such as role play^34^, imagery exposure^35^ and rescripting therapy^36^. In these contexts, mental imagery is commonly deployed in an intentional, goal-directed way, in which individual attempts to imagine specific content, guided by their own intentions or with the assistance of an external guide (e.g., a game master, officiant, or therapist)^23,37–40^. However, the effectiveness of mental imagery in such settings may depend on an individual’s ability to alter the vividness and emotional sentiment of the imagined content^35,40^. External stimuli capable of influencing mental imagery, such as music, are therefore of great interest in such contexts.

More than 70% of participants report commonly experiencing mental imagery whilst listening to music^41–43^. Such imagery contains both realistic (e.g., images from past events and landscapes) as well as abstract (e.g., colours and shapes) imagined content^41^. Moreover, music influences vividness, spatial–temporal, and emotional sentiment, specifically in deliberate, goal-directed mental imagery^14^, with eye closure intensifying these effects^44,45^. Findings further suggest that music, compared to silence, evokes more extended narratives, and predicts imagery with greater affect, confidence, and social dynamics^46–48^. Yet, a deep understanding of the effects of specific musical features on mental imagery^9,14,22^ remains unclear.

Some previous studies observed that tempo can act as a mediator, where faster tempo in a directed mental imagery paradigm predicted less imagined distance travelled and time passed, as well as greater positive sentiment^14^. Similarly, decreased mind-wandering and increased meta-awareness were associated with faster tempo in an unintentional mind-wandering task^13^. Musical structure manipulations and thematic entrances can also manipulate the subjective experience of imagined episodes, often eliciting positive emotions and relaxation, particularly during states of absorption^8,10,49^. Recently, Jakubowski et al.^9^ scrutinised how music-evoked thoughts—ranging from sensory experiences (e.g., olfactory, gustatory) to autobiographical memories and fictional stories—are influenced by musical genre, emotional expression, familiarity and liking. They found that classical and electronic music elicited more thoughts than pop or rock music, with genre, valence, and familiarity shaping the occurrence, type, and novelty of these thoughts. It has also been demonstrated that systematic changes in musical parameters, such as pitch contour, dynamics, and tonal stability, can elicit distinct spatial and motion-related imagery, showing the potential role of cross-modal correspondences^50–53^. These findings illustrate the potential of specific musical features to affect mental imagery. Crucially, however, much of the existing research has focused on fully orchestrated Western musical stimuli (e.g^13,14,54^.,). This is important to consider, as music-evoked mental imagery may vary between individuals depending on cultural background^3^, musical listening habits^55^, or musical expertise^56^. For example, imagined content is more similar within cultures than between^3^, and prevalence of music-evoked mental imagery rises during absorbed music listening^57^. In addition, fully orchestrated music renders isolating the effects of individual features difficult, highlighting the need for studies to systematically explore and vary specific musical features to better understand their effects on mental imagery.

A bottom-up exploration of features implicated in music-evoked mental imagery should consider existing music that has already been used to support processes akin to mental imagery. For example, multiple cultures or traditions, such as Mandé culture of West Africa, deploy ritual repetitive drumming to support catharsis (e.g., ritual participants releasing negative emotions)^23,58^. The ‘healer’ in these rituals guides participants to evoke various forms of mental images, such as memories and scenarios, which may in turn influence their emotional states, while listening to or participating in musical activity^25^. This approach is somewhat similar to modern imagery-based therapies^23^, such as Imagery Rescripting and Imaginal Exposure^35,39,59^. Ethnomusicologists studying these types of traditional ritual music emphasise that they often focus on repetitive quasi-isochronous patterns—beats with nearly equal, but not perfectly uniform, intervals between sound onsets^1,58^—played with expressive micro-variations rather than mechanically identical rhythms^28,58,60,61^. Mechanical here refers to a theoretical performance style whereby each drum strike is perfectly identical to the previous one in terms of timing, velocity, and strike location, without any small intentional or unintentional variations between strikes and timbre^26,62,63^. In contrast, expressive, deliberate micro-variations in timing (typically less than 50 ms)^60^, dynamics, and articulation in performance can convey emotion, expression, and interpretation^64^, and have been shown to support synchronisation of sensory and motor activities with rhythmic cues^65^. This provides a foundation for further exploration of whether and how micro-variations can influence higher-order processes like mental imagery likely due to their emotionally evocative nature, consistent with findings that emotion and visual imagery are closely related^22^.

Regarding micro-variations in timing and loudness, timing variations as small as 15 ms, can affect listeners’ aesthetic preferences (e.g., liking^66^) and musical traditions of expressive micro-variations can communicate group cohesion and cultural origin through them^1,27,28,64^. Similarly, studies have shown that small-scale variability in drum strike velocity, such as variations influenced by strike force and starting height, can enhance expressive depth and variability in drumming^67^, with expert drummers routinely demonstrating superior precision and control in their strike velocity variability compared to amateurs^68,69^*.* Besides intentional, expressive micro-variations, musical performances by humans also contain random micro-variations, which arise from the intrinsic randomness involved in movement generation and attention^70,71^. These variations are not deliberately controlled by the performer, nor are they entirely accidental; instead, they emerge naturally and are coupled with physiological constraints such as muscle fatigue or refractory periods^72^, and external noise sources^73^. Whilst it is easy to dismiss unintentional random micro-variation as an undesired source of error in performance, these human random micro-variations between strikes and timbre play an important role in contributing to naturalness (i.e., lifelike, organic, and authentic, rather than mechanical) and human-sounding performances. Indeed, a study demonstrated that listeners robustly prefer these musical rhythms with human-like long-range correlated fluctuations over perfectly timed beats^74^.

The evidence to date suggests that random micro-variations significantly influence cognitive and perceptual processes^60,64,75,76^; however, their effect on mental imagery is unknown. The objective of this study was to understand how random micro-variation, as opposed to mechanical playing, affects mental imagery characteristics in a directed mental imagery paradigm^14^. Participants performed a mental imagery task with their eyes closed in which they were instructed to imagine a continuation of a journey prompted by a visual inducer. After each trial, participants completed questions regarding vividness, distance travelled and time passed of the imagined content, and provided an open-text response describing their experience. We additionally collected data on individuals’ proneness to imaginative and altered states (absorption), their ability to imagine vividly, and level of musical training, in order to control for these factors when analysing the results. Whilst performing the task, participants either listened to silence, mechanical drum patterns, or drum patterns containing small random micro-variations. We used quasi-isochronous drum patterns played at 240 beats per minute (BPM), which is similar to the shamanic ritual repetitive drumming observed by several interdisciplinary researchers^77–82^. Each of our 100 participants listened to 7 stimuli in a fully random order: the quasi-isochronous drum pattern in the three timbres (i.e., instruments), each once mechanical and once with micro-variations. To avoid simply replicating music production effects such as the ‘machine gun’ effect^83^, the auditory stimuli were generated using physical models of drums^84^ that are struck with simulated strikes. This approach inherently introduces a small amount of randomisation to the timbre, even in the mechanical condition, preventing the repetitive and unnatural sound typical of the machine-gun effect. A physical drum model, rather than a sample-based sequencer, has the advantage that, when modelling mechanically identical strikes, the resulting sounds differ slightly with each strike due to the ongoing and complex vibrations of the drum from previous strikes. In the micro-variation condition, additional minor random variations in terms of timing, strike velocity, and drum-hit location were applied to the strikes, resulting in a more “human-like” performance.

To summarise, we investigate the influence of micro-variations on directed mental imagery. Based on previous research and theoretical frameworks, we test the following hypotheses:

1. Random micro-variations induce more imagined vividness than mechanical playing and silence (supported)
2. Random micro-variations induce more imagined positive emotional sentiment than mechanical playing and silence (not supported)
3. Auditory conditions induce greater imagined travelled distance and time than silence (supported for imagined distance, partially supported for imagined time)
4. Interactions between conditions and individual differences in imagery characteristics were investigated in an exploratory fashion.

## Results

### Random micro-variations induce more vivid imagery than silence and mechanical playing

**Fig. 1:**
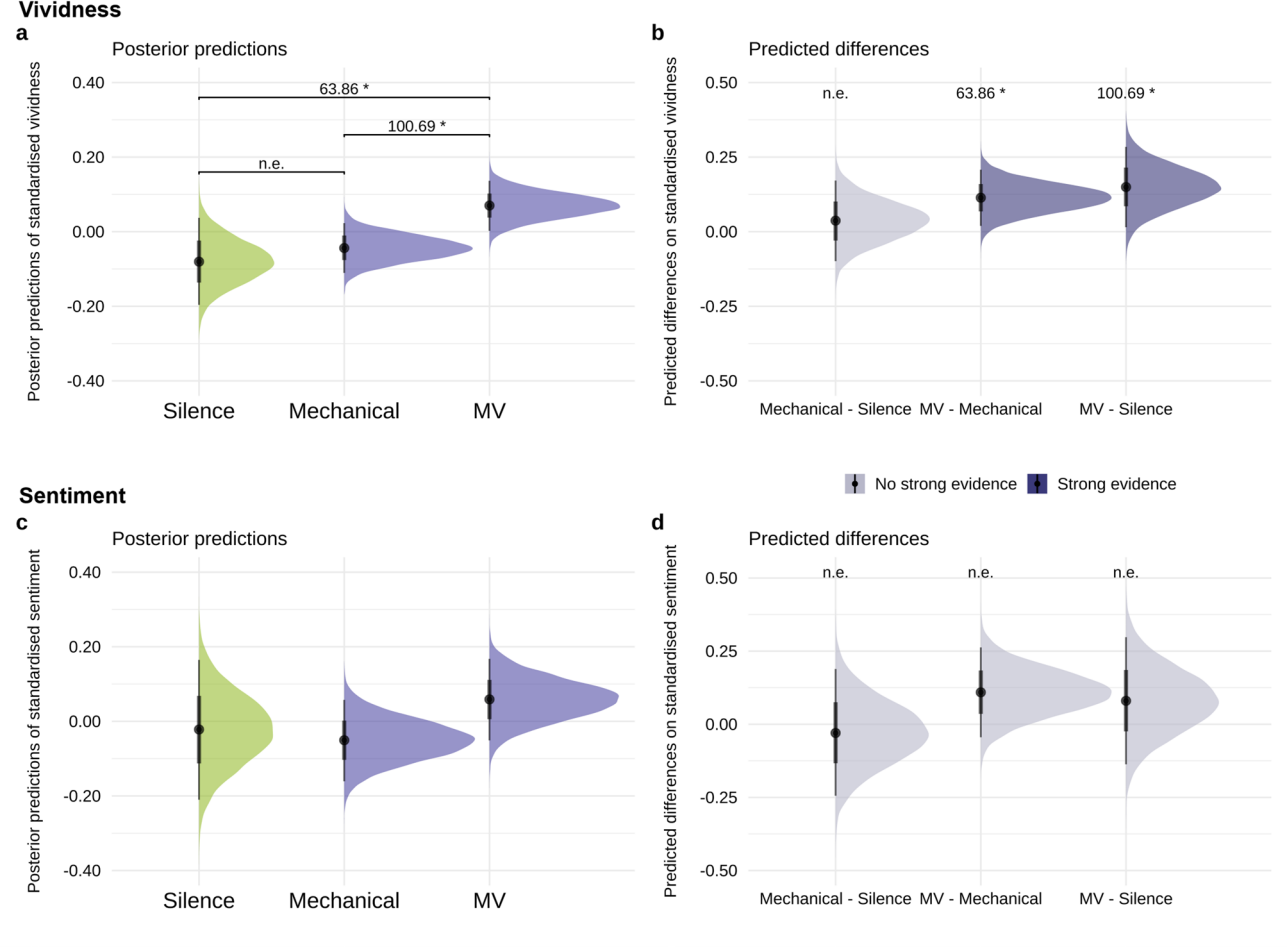
Posterior predictions of standardised Vividness and Sentiment in the Silence, Mechanical, and Micro-Variation (MV) conditions. The left column (**a, c**) shows the model’s posterior predictions of (**a**) vividness and (**c**) sentiment for the conditions, and the right column (**b, d**) shows predicted differences of (**b**) vividness and (**d**) sentiment between the conditions. For predicted differences, dark purple indicates strong evidence, whereas light purple indicates no strong evidence. “n.e.” corresponds to “no strong evidence”. Error bars show 95% CI. *Odds* ≥ 19 are strong evidence for one-sided hypothesis tests, indicated above the parentheses with an evidence ratio and marked with an asterisk (*).

Participants used the entire range of the 100-point vividness scale, with responses spanning from 0 to 100 (*M* = 51.42, *SD* = 27.65). Bayesian multilevel regression model predicted standardised vividness based on auditory condition (factor with three levels: Silence, Mechanical, and Random Micro-Variation) as a predictor, whilst controlling for participant and trial number (more details of the model in the Method section). As shown in Fig. 1a,b, we observed strong evidence (*Odds* ≥ 19 for one-sided hypothesis tests^85^) that playing drum patterns with random micro-variation increased vividness of mental imagery compared to silence (*β* = 0.15, *EEβ* = 0.07, *Odds* (*β* > 0) = 63.86*, *Post.Prob* = 0.98) as well as mechanical playing (*β* = 0.11, *EEβ* = 0.05, *Odds* (*β* > 0) = 100.69*, *Post.Prob* = 0.99). There was no compelling difference in vividness between silence and mechanical playing (*Odds* = 2.36, *Post.Prob* = 0.70).

### Random micro-variations show no strong evidence on imagined emotional sentiment compared to silence, but weak to moderate evidence compared to mechanical playing

Imagined sentiment, referring to the emotional tone attributed to participants’ mental imagery episodes, was obtained from the open-text responses describing their imagery. NTLK^86^ and VADER^87^ were used to analyse sentiment by giving numerical scores from negative to positive for each description. They estimate emotional valence and intensity on a continuous scale, with higher values reflecting more positive sentiment. We observed no evidence that patterns with random micro-variation increased sentiment compared to the silence control (*Odds* = 3.31, *Post.Prob* = 0.77) and weak to moderate of an increase compared to the mechanical style (*β* = 0.11, *EEβ* = 0.08, *Odds* (*β* > 0) = 11.49, *Post.Prob* = 0.92). Additionally, we observed no strong evidence for a predictive relationship between silence and mechanical conditions (*Odds* = 0.03, *Post.Prob* = 0.40; Fig. 1c,d).

### Random micro-variations show very strong evidence on imagined distance travelled compared to silence, and weak to moderate evidence compared to mechanical playing

**Fig. 2:**
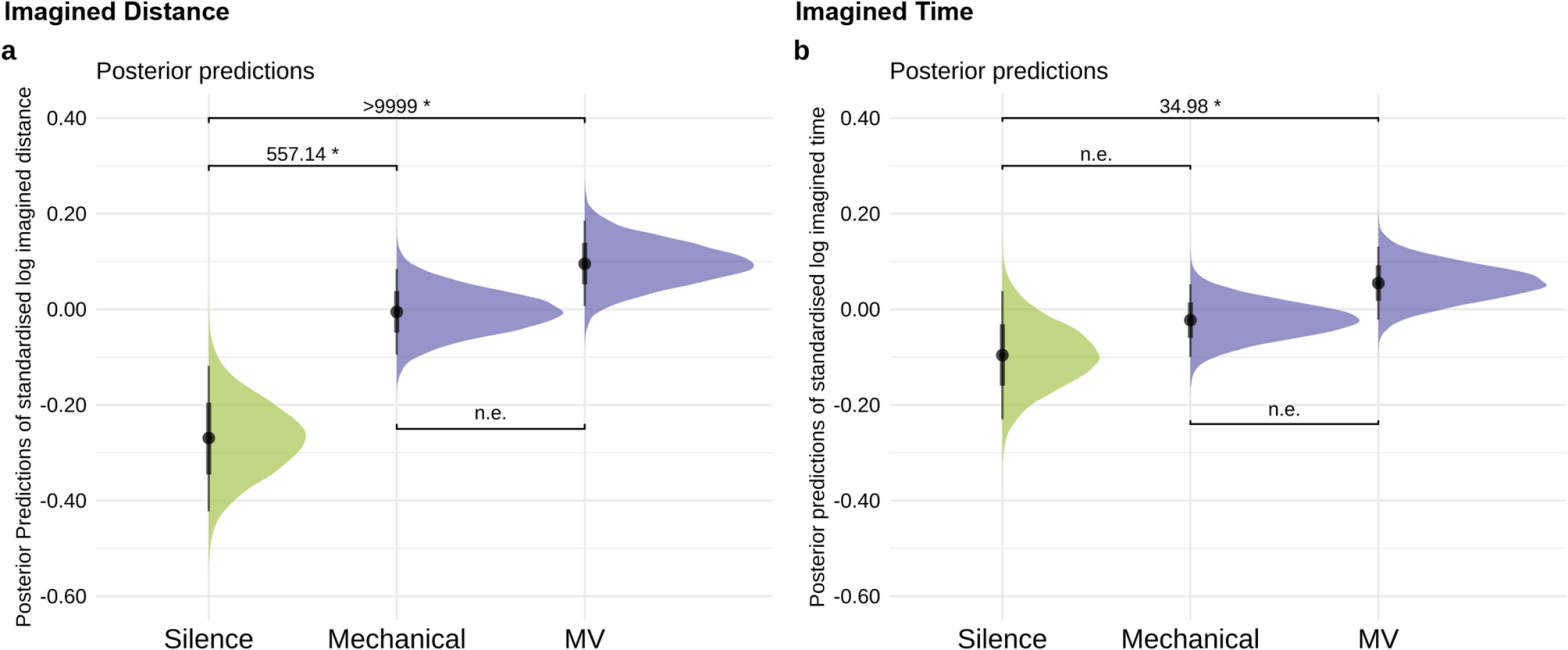
Posterior predictions of the standardised and log-transformed Imagined Time and Distance in Silence, Mechanical, Micro-Variation (MV). The left column (**a**) shows the model’s posterior predictions of imagined distance, and the right column (**b**) column shows the model’s posterior predictions of imagined time for the conditions. Error bars show 95% CI. “n.e.” corresponds to “no strong evidence”. *Odds* ≥ 19 are deemed strong evidence for one-sided hypothesis tests, indicated above the parentheses with an evidence ratio and marked with an asterisk (*).

We observed very strong evidence that in both drumming conditions, participants imagined greater distances travelled compared to the silence condition (random micro-variations vs silence: *β* = 0.37, *EEβ* = 0.09, *Odds* (*β* > 0) > 9999*, *Post.Prob* = 1.00, mechanical vs silence: *β* = 0.26, *EEβ* = 0.09, *Odds* (*β* > 0) = 557.14*, *Post.Prob* = 1.00). There was only weak to moderate evidence for a difference between random micro-variation and mechanical condition (*Odds* = 16.43, *Post.Prob* = 0.94). See Fig. 2b for an illustration of posterior predictions of these findings.

### Random micro-variations predict longer imagined time travelled than silence, but show no strong evidence compared to mechanical playing

Compared to the silence condition, we observed strong evidence that the imagined time travelled was greater in the random micro-variation condition (*β* = 0.15, *EEβ* = 0.08, *Odds* (*β* > 0) = 34.98*, *Post.Prob* = 0.97). However, there were no compelling differences between mechanical and silence (*Odds* = 4.69, *Post.Prob* = 0.82) as well as micro-variation and mechanical conditions (*Odds* = 11.42, *Post.Prob* = 0.92). See Fig. 2a for an illustration of posterior predictions of these findings.

### Exploring individual differences in mental imagery across conditions

We also analysed the effects of absorption tendencies (measured by the “prone to imaginative and altered states” subscale of Tellegen Absorption Scale (TAS)^88^), vividness of visual imagery tendencies (measured by Vividness of Visual Imagery Questionnaire (VVIQ)^89^), and Musical Training (measured by the “musical training” (MT) subscale of Goldsmiths Musical Sophistication Index (GMSI)^90^) with each condition, using one-sided hypothesis tests (*Odds* ≥ 19 for strong evidence)^85^. High (*Mean* + 1*SD*) and low (*Mean *- 1*SD*) scores were defined as one standard deviation above or below the mean. Additionally, we examined their interactions with the silence, mechanical and random micro-variation conditions, on vividness, sentiment (VADER), imagined distance, and imagined time, applying two-sided hypothesis tests (*Odds* ≥ 39 for strong evidence)^91^.

#### Vividness

##### TAS

**Fig. 3:**
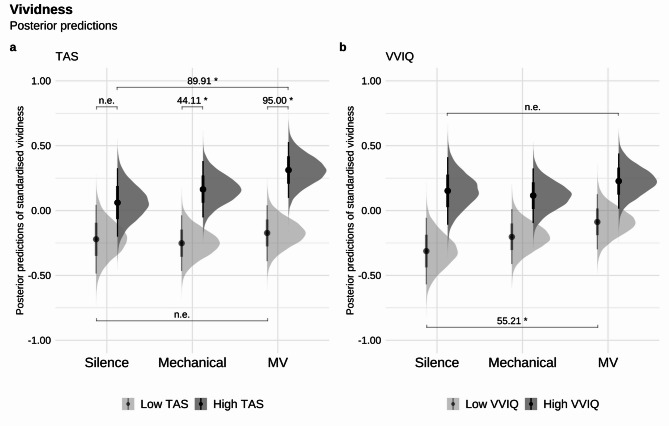
Posterior predictions of the Standardised Vividness in Silence, Mechanical, Micro-Variation (MV), and whether the predictions differ based on Tellegen Absorption Scale (TAS, in *SD*), and Vividness of Visual Imagery Questionnaire (VVIQ, in *SD*) in two levels [high (TAS = *Mean* + 1*SD*; VVIQ = *Mean* + 1*SD*) and low (TAS = *Mean *- 1*SD*; VVIQ = *Mean *- 1*SD*)]. The left column shows the model’s posterior predictions of Vividness by (**a**) TAS level, and the right column shows the model’s posterior predictions of Vividness by (**b**) VVIQ level. Darker grey indicates high scale level, and lighter grey indicates low scale level. Error bars show 95% CI. “n.e.” corresponds to “no strong evidence”. *Odds* ≥ 19 are deemed strong evidence for one-sided hypothesis tests and *Odds* ≥ 39 are deemed strong evidence for two-sided hypothesis tests, indicated above the parentheses with an evidence ratio and marked with an asterisk (*).

There was strong evidence that higher trait level absorption scores on the TAS in general are associated with increased vividness of the imagined content (*β* = 0.43, *EEβ* = 0.20, *Odds* (*β* > 0) = 63.00*, *Post.Prob* = 0.98). However, when broken down by condition, this effect was only observed in the random micro-variation (*β* = 0.49, *SE* = 0.21, *Odds* (*β* > 0) = 95.00*, *Post.Prob* = 0.99) and mechanical conditions (*β* = 0.42, *SE* = 0.21, *Odds* (*β* > 0) = 44.11*, *Post.Prob* = 0.98), but not in the silence condition (*Odds* = 7.43, *Post.Prob* = 0.88). When compared directly with the silence condition, only the random micro-variation (*β* = 0.25, *SE* = 0.11, *Odds* (*β* > 0) = 89.91*, *Post.Prob* = 0.99), but not the mechanical condition (*Odds* = 4.85, *Post.Prob* = 0.83), yielded compelling evidence for a greater effect of individuals with higher absorption tendencies on vividness. There was no strong difference between the random micro-variation and mechanical conditions (*Odds* = 36.27, *Post.Prob* = 0.97) in individuals with higher absorption tendencies on vividness. For individuals with lower absorption tendencies, vividness scores were consistently and comparably low across all conditions, with no evidence of a strong difference between any specific condition (all *Odds* ≤ 5.52, all *Post.Prob* ≤ 0.85, see Fig. 3a).

##### VVIQ

There was also strong evidence that higher vivid imagery tendencies on VVIQ are associated with increased vividness of the imagined content (*β* = 0.34, *EEβ* = 0.19, *Odds* (*β* > 0) = 22.98*, *Post.Prob* = 0.96). Those with only lower vivid imagery tendencies showed a stronger effect on vividness in the random micro-variation compared to the silence condition (*β* = 0.22, *EEβ* = 0.11, *Odds* (*β* > 0) = 55.21*, *Post.Prob* = 0.98, see Fig. 3b).

##### MT

We observed no strong evidence that musical training scores influenced vividness across conditions (all *Odds* ≤ 23.17, all *Post.Prob* ≤ 0.96).

#### Emotional sentiment

##### TAS. VVIQ. MT

We observed no strong evidence that absorption tendencies (all *Odds* ≤ 7.04, all *Post.Prob* ≤ 0.88), vividness of visual imagery tendencies (all *Odds* ≤ 6.62, all *Post.Prob* ≤ 0.87), and musical training scores (all *Odds* ≤ 6.72, all *Post.Prob* ≤ 0.87) influenced sentiment across conditions.

#### Imagined distance

##### TAS

**Fig. 4:**
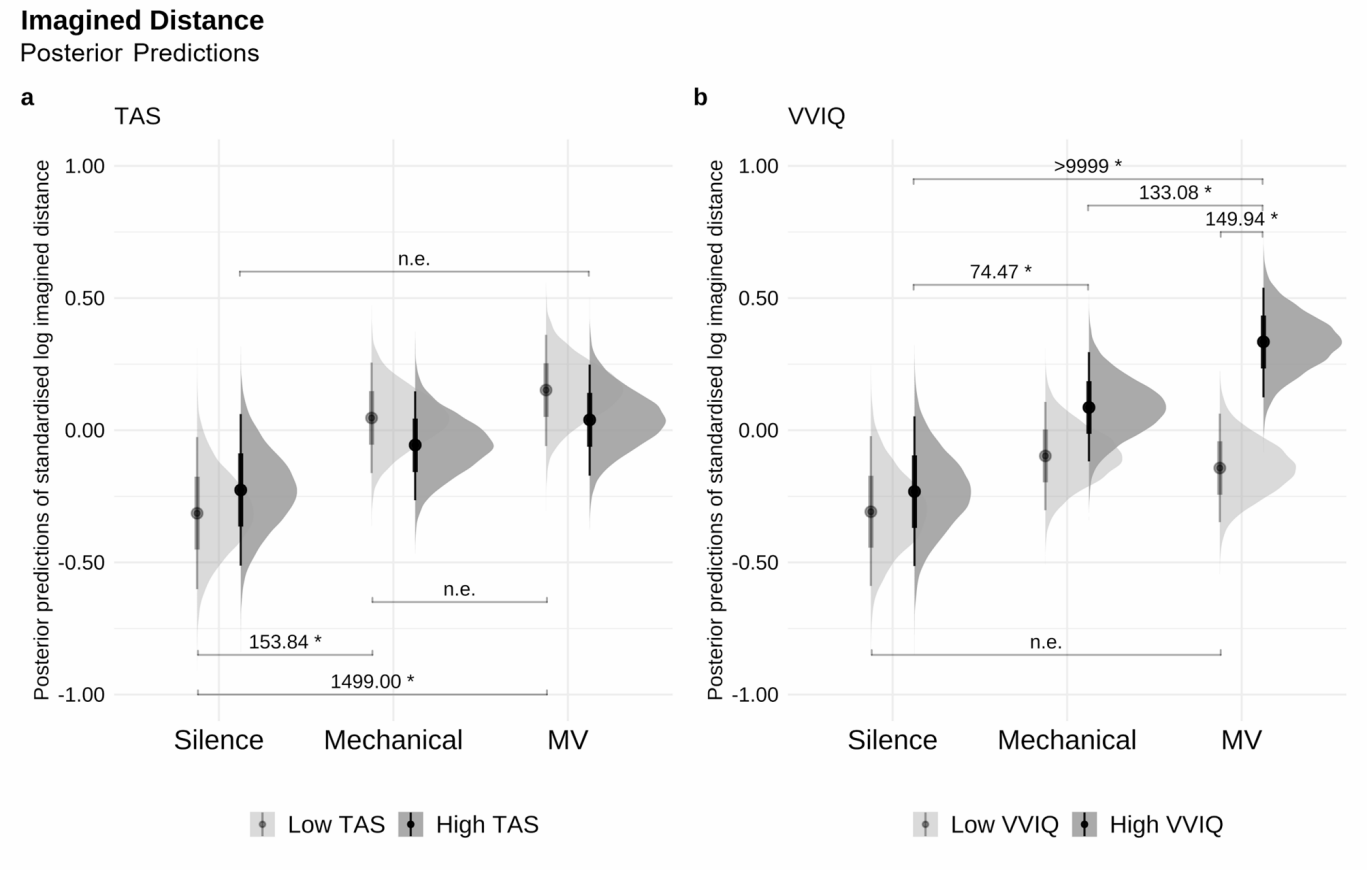
Posterior predictions of the standardised and log-transformed Imagined Distance in Silence, Mechanical, Micro-Variation (MV), and whether the predictions differ based on Tellegen Absorption Scale (TAS, in *SD*), and Vividness of Visual Imagery Questionnaire (VVIQ, in *SD*) in two levels [high (TAS = *Mean* + 1*SD*; VVIQ = *Mean* + 1*SD*) and low (TAS = *Mean *- 1*SD*; VVIQ = *Mean *- 1*SD*)]. The left column shows the model’s posterior predictions of Imagined Distance by (**a**) TAS level, and the right column shows the model’s posterior predictions of Imagined Distance by (**b**) VVIQ level. Darker grey indicates high scale level, and lighter grey indicates low scale level. Error bars show 95% CI. “n.e.” corresponds to “no strong evidence”. *Odds* ≥ 19 are deemed strong evidence for one-sided hypothesis tests and *Odds* ≥ 39 are deemed strong evidence for two-sided hypothesis tests, indicated above the parentheses with an evidence ratio and marked with an asterisk (*).

There was no strong evidence that higher absorption scores on the TAS were associated with heightened imagined distance on the experimental task (*Odds* = 0.47, *Post.Prob* = 0.32). Although higher absorption individuals demonstrated no strong evidence across conditions (*Odds* = 26.49, *Post.Prob* = 0.96), individuals with lower absorption tendencies were associated with stronger effects on imagined distance in the random micro-variation (*β* = 0.47, *SE* = 0.14, *Odds* (*β* > 0) = 1499.00*, *Post.Prob* = 1.00) and mechanical style (*β* = 0.36, *SE* = 0.14, *Odds* (*β* > 0) = 153.84*, *Post.Prob* = 0.99), both relative to silence (Fig. 4a).

##### VVIQ

Overall, individuals with more vivid visual imagery scores on the VVIQ did not provide strong evidence for greater levels of imagined distance on the experimental task (*β* = 0.29, *SE* = 0.17, *Odds* (*β* > 0) = 23.49, *Post.Prob* = 0.96). Yet, when split by condition, a relationship was observed only in the random micro-variation (*β* = 0.48, *SE* = 0.19, *Odds* (*β* > 0) = 149.94*, *Post.Prob* = 0.99), but not in the mechanical (*Odds* = 5.02, *Post.Prob* = 0.83) nor silence (*Odds* (*β* > 0) = 1.68, *Post.Prob* = 0.63) conditions. When micro-variation condition was directly compared with the mechanical condition, higher vivid imagery tendencies were associated with greater imagined distance travelled (*β* = 0.25, *SE* = 0.10, *Odds* (*β* > 0) = 133.08*, *Post.Prob* = 0.99). A stronger effect was observed for individuals with higher vivid imagery tendencies on imagined distance in both random micro-variation (*β* = 0.57, *SE* = 0.14, *Odds* (*β* > 0) > 9999*, *Post.Prob* = 1.00) and mechanical style (*β* = 0.32, *SE* = 0.14, *Odds* (*β* > 0) = 74.47*, *Post.Prob* = 0.99) conditions compared to the silence condition. There was no strong evidence for lower vivid imagery tendencies across conditions (all *Odds* ≤ 12.99, all *Post.Prob* ≤ 0.93; Fig. 4b).

##### MT

**Fig. 5:**
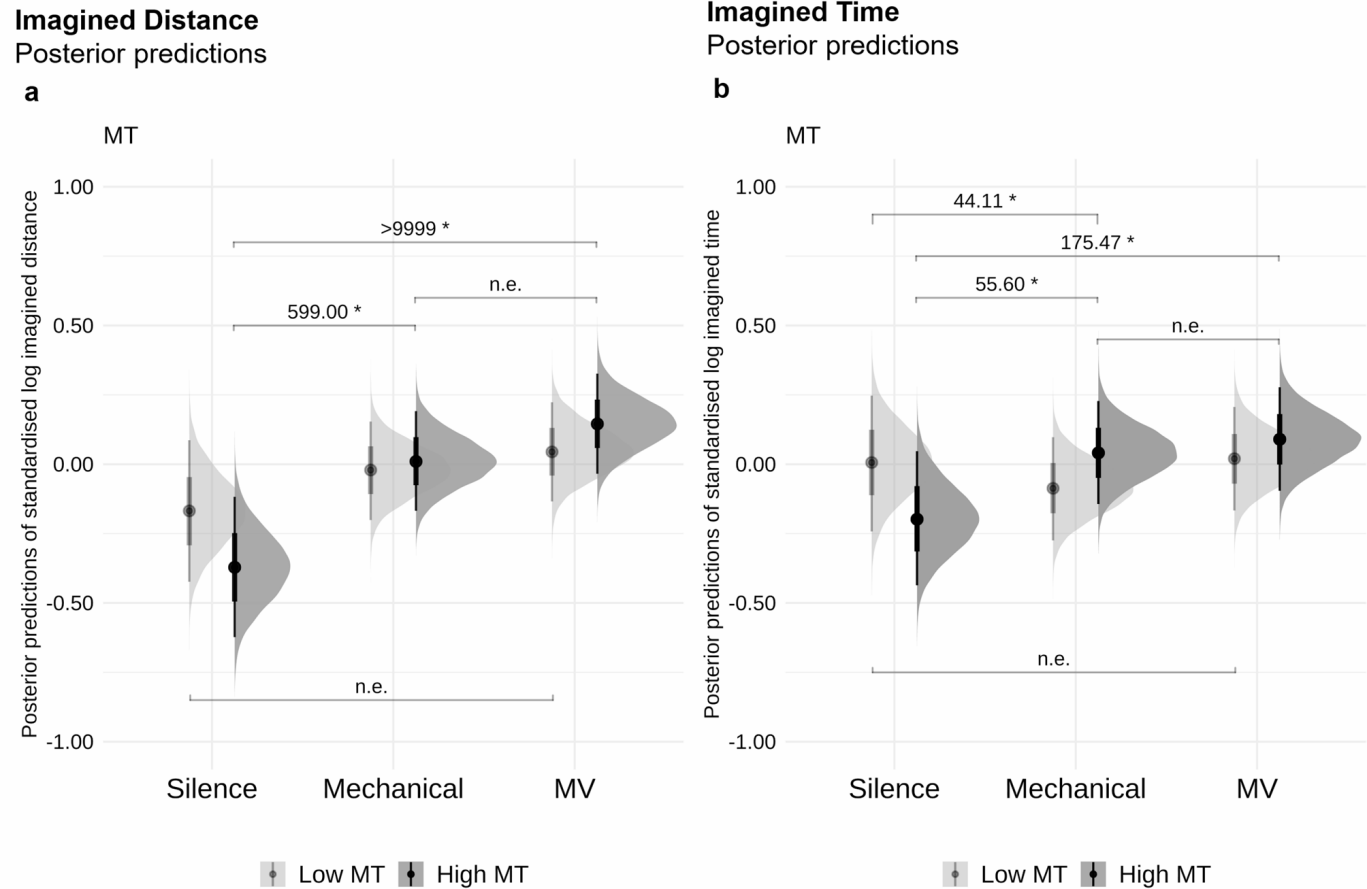
Posterior predictions of the standardised and log-transformed Imagined Distance and Time in Silence, Mechanical, Micro-Variation (MV), and whether the predictions differ based on Musical Training (MT in GMSI, in *SD*) scores in two levels [high (GMSI = *Mean* + 1*SD*) and low (MT = *Mean *- 1*SD*)]. The left column shows the model’s posterior predictions of (**a**) Imagined Distance by MT level, and the right column shows the model’s posterior predictions of (**b**) Imagined Time by MT level. Darker grey indicates high scale level, and lighter grey indicates low scale level. Error bars show 95% CI. “n.e.” corresponds to “no strong evidence”. *Odds* ≥ 19 are deemed strong evidence for one-sided hypothesis tests and *Odds* ≥ 39 are deemed strong evidence for two-sided hypothesis tests, indicated above the parentheses with an evidence ratio and marked with an asterisk (*).

There was no strong evidence that higher musical training scores were associated with greater imagined distance on the task (*Odds* = 1.36, *Post.Prob* = 0.58). Higher levels of musical training were associated with stronger effects on imagined distance in the random micro-variation (*β* = 0.52, *SE* = 0.13, *Odds* (*β* > 0) > 9999*, *Post.Prob* = 1.00) and mechanical style (*β* = 0.38, *SE* = 0.13, *Odds* (*β* > 0) = 599.00*, *Post.Prob* = 1.00) compared to silence. However, there were no compelling differences between random micro-variation and mechanical conditions (*Odds* = 12.61, *Post.Prob* = 0.93) on imagined distance. There was also no strong evidence for lower musical training scores across conditions (*Odds* = 18.59, *Post.Prob* = 0.93; Fig. 5a).

#### Imagined time

##### TAS. VVIQ

We found no strong evidence that absorption tendencies (all *Odds* ≤ 26.30, all *Post.Prob* ≤ 0.96) and vividness of visual imagery tendencies (all *Odds* ≤ 15.42, all *Post.Prob* ≤ 0.94) influenced imagined time across conditions.

##### MT

There was no compelling evidence to suggest that overall higher musical training scores are generally associated with increased imagined time (*Odds* = 1.74, *Post.Prob* = 0.64). However, when split by conditions, individuals with higher musical training in the random micro-variation (*β* = 0.29, *SE* = 0.11, *Odds* (*β* > 0) = 175.47*, *Post.Prob* = 0.99) and mechanical (*β* = 0.24, *SE* = 0.11, *Odds* (*β* > 0) = 55.60*, *Post.Prob* = 0.98) conditions showed a longer imagined time travelled compared to silence. Meanwhile, there was no compelling difference between random micro-variation and mechanical style (*Odds* = 2.69, *Post.Prob* = 0.73). There was also no strong effect for individuals with lower musical training on imagined time across all conditions (all *Odds* ≤ 10.19, all *Post.Prob* ≤ 0.91; Fig. 5b). Furthermore, the difference in imagined time between the silence and mechanical conditions increased with increasing musical training (*β* = 0.33, *SE* = 0.16, *Odds* (*β* < 0) = 44.11*, *Post.Prob* = 0.98).

## Discussion

This study investigated how random micro-variations, a core component of human music performance, in quasi-isochronous repetitive drumming patterns affect the vividness, emotional sentiment, as well as imagined distance and time travelled of the imagined content in concurrent directed mental imagery. Our findings reveal that quasi-isochronous drumming with random micro-variation increases vividness of imagined content compared to silence. There is compelling evidence that drumming with micro-variation enhances vividness of imagined content more than purely mechanical drumming. Furthermore, random micro-variations – but not mechanical performances – also induce longer imagined time of travel than silence, and both random micro-variation and mechanical conditions lead to greater imagined distance travelled than silence. We also found that individual differences, such as tendencies toward absorption and imagining vividly, and musical training, interact with drumming to shape mental imagery. The pattern of results, as well as their potential recreational, aesthetic, and clinical implications and future directions are discussed in the following.

Our research demonstrates that auditory stimulation can influence the vividness of mental imagery compared to silence, aligning with previous reports^14,44,46,48^. Our most striking finding is that random micro-variation enhanced the vividness of imagined content more than mechanical drumming. We speculate that one possible explanation for this finding is that listeners may perceive drumming with micro-variation as more natural and organic by listeners, potentially aligning with a general preference for such rhythms in mental imagery. While the role of this preference in mental imagery remains uncertain, prior work suggests that the inherently human quality of these variations evokes a sense of authenticity and pleasantness absent from mechanical performances^74^. Moreover, vividness of imagined content seems to be associated with the aesthetic appeal of music, although this appeal varies from individual to individual^15^. In addition to aesthetic and perceptual speculation, alternative explanations could come from embodied cognition frameworks, where listeners internally simulate action-related qualities of the sounds, that may enrich the vividness of mental imagery. Findings that human-performed music enhances sensorimotor and synesthetic responses more than a “deadpan” (without expression) performance^92^ support this position. Accordingly, such embodiment engagement may contribute to richer internal representations potentially explaining why micro-variation enhances vividness of imagined content more than mechanical condition. This is also especially relevant in the context of drumming, where it is the highly physical form of music-making where sound is directly shaped by movement, and thus the expressivity^93^. Taken together, the current paper’s finding highlights random micro-variation as a distinct musical feature capable of enhancing the vividness of mental imagery.

The results showed no strong evidence for emotional sentiment being affected by random micro-variations and mechanical playing, compared to silence. This is an important observation as it supports prior findings that the effect of music on vividness and sentiment of imagined content can be distinct^14^. Furthermore, the finding suggests that random micro-variation, as a specific musical feature, have a targeted effect, influencing vividness of imagined content while having minimal to no impact on emotional sentiment. This distinction allows for a degree of dissociation between these processes, providing valuable insights into how specific musical features uniquely contribute to different aspects of mental imagery. One potential explanation for the lack of strong emotional effects is the repetitive rhythmic nature of our stimuli and the use of micro-timing variations with a standard deviation as small as 4 ms. These patterns, while ideal for isolating micro-variation effects, may not engage emotional processing as effectively as musical stimuli with richer rhythmic, melodic, and harmonic content. Previous studies have shown that structural features such as harmony and rhythm evoke strong neural engagement in emotion-related areas of the brain (e.g., limbic system)^94,95^. Alternatively, or in addition, the effect of random micro-variation should be understood as enhancing existing effects of the musical stimuli. Since quasi-isochronous drum patterns do not evoke strong emotions to begin with, random micro-variations may have little sentiment to affect.

Both random micro-variation and mechanical drumming elicited greater imagined distance compared to silence. This is consistent with prior studies also observing greater imagined distance travelled during music listening^14,44^. While individuals typically maintain awareness of their physical surroundings and the present moment^96^, spatial navigation enables the formation of mental representations linking different points and allows behavioural flexibility for goal-directed planning^97^. Drumming likely served as an attentional anchor^98^, shaping mental representations and navigational cues. The repetitive patterns of drumming may have enhanced continuity in imagined distance. As the hierarchical structure of the musical stimuli used here are not rich, the attention may have shifted to micro-variations, such as micro-timing (as hypothesised by Margulis^99^). Micro-timing changes, which have been associated with increased mental effort (as indicated by pupillometry)^75^, might have anchored attention, thereby contributing to the perception of greater imagined distance.

Interestingly, for imagined time, only random micro-variation showed stronger evidence for longer imagined time passed compared to silence, whereas the mechanical condition did not show strong evidence for a difference. This is somewhat unexpected, particularly given previous findings that even repetitive, mechanical sounds can influence perceived time through entrainment (i.e., the synchronisation of cognitive rhythmic processes with external rhythmic stimuli)^76,100,101^. In our model, the posterior probability of a positive effect of the mechanical condition on imagined time (relative to silence) was 0.82, which is not sufficient to draw firm conclusions. If there is indeed no or only a small effect of mechanical rhythms on imagined time, one possible explanation could be that participants habituated to their high predictable pattern^102^, and that individuals may not require as much cognitive effort or flexibility to monitor time. In turn, this could result in a perception of time that feels static or unchanging, similar to the experience during silence. Specifically, repeated stimuli are perceived to last for shorter durations in contrast to novel stimuli, potentially because neural responses diminish with repeated exposure^103^. Another possibility is that the visual imagery task, which is based on imagining physical distance, may have dominated participants’ temporal judgments, thereby attenuating subtle entrainment effects associated with the mechanical condition. Perhaps future research could better disentangle this effect by using alternative control conditions, such as static visuals, to isolate modality-specific contributions. In contrast, engaging activities with variability and complexity often make time seem to pass more quickly, a phenomenon commonly referred to as “time flies when you’re having fun”^104^. The randomness of micro-variations, such as in time and loudness, might create more moments of novelty, increasing the level of engagement^105^.

### Individual differences

There is significant variation among individuals in their capacity to form mental images^106^. First considering the absorption level on the TAS, our findings showed that individuals with higher absorption tend to experience more vivid mental imagery in both random micro-variation and mechanical conditions, but not in the silence condition. This effect was particularly pronounced in the random micro-variation condition. Interestingly, however, level of absorption was not found to exert the same effect for emotional sentiment. These findings suggest that while individuals higher in absorption are more likely to deeply engage with auditory stimuli, especially random micro-variations, and vividly imagine in response to them, this heightened absorption does not necessarily translate to stronger imagined emotional content. Initially, it might be puzzling why in terms of vividness, absorption shows such a clear effect in both music conditions, but not the silence condition. It might be that individuals with higher propensity for absorption benefit from the additional mental imagery support that music—remarkably random micro-variations—provides, as evidenced by their increased vividness scores in music conditions compared to silence. This suggests that absorption tendency may predict an individual’s capacity to engage with music to enhance mental imagery.

Our findings also revealed that individuals with lower absorption tendencies experienced greater imagined distance in both random micro-variation and mechanical conditions compared to silence. Conversely, no strong association emerged for individuals with higher absorption on imagined distance. This suggests that subtle variations within quasi-isochronous patterns may provide external auditory cues that facilitate spatial exploration for individuals with lower absorption tendencies, who may otherwise rely less on internal mental imagery. Integrating these findings with vivid visual imagery tendencies, as measured by the VVIQ, revealed complementary insights. Individuals with higher vivid imagery tendencies imagined greater distances on the task, most pronounced in the micro-variation condition. For individuals with lower absorption tendencies, quasi-isochronous rhythms with micro-variation and mechanical style were associated with increased imagined distance. Meanwhile, higher vivid imagery tendencies seem to align more directly with the ability to engage in spatial exploration (e.g., to reach a goal, i.e., the landmark in the visual stimuli^97^). Taken together, these results indicate that individual differences in absorption and imagery vividness shape how simplistic auditory structure supports imagined movement either by compensating for weaker internal imagery in low absorbers and/or by enhancing already vivid mental representations in high visualisers.

Relating to musical training as an interacting effect for mental imagery in this study, it is also worth noting that as musical expertise increased, participants imagined greater distances travelled and longer times spent in micro-variation and mechanical conditions than in the silence condition. Furthermore, and again, micro-variation had a more pronounced effect than mechanical style. The observed effect on imagined time in both the micro-variation and mechanical conditions suggests that their shared rhythmic predictability engages the temporal imagery of highly trained listeners in similar ways, resulting in no strong difference between the two conditions. A similar pattern was observed for imagined distance, suggesting that as musical expertise increases, rhythmic auditory input may serve as a stronger cue for constructing spatiotemporal imagery, while silence may result in a lower baseline. Considering that micro-variations had a larger overall effect on this variable, this study is the first, among those employing this mental imagery paradigm^14,44,46,48,107^, to understand how specific musical mechanisms – namely, micro-variations – interact with musical training to influence imagined distance and time.

### Implications and future directions

The present findings support music’s potential to play a beneficial role in evidence-based therapies^108^, creative practices^109^, as well as recreational settings that utilise mental imagery^39^. Quasi-isochronous patterns with random micro-variations could be applied in such settings to enhance the vividness of individuals’ imagined scenarios as well as spatiotemporal properties, both independent of the emotional sentiment of imagined content. For example, individuals suffering from Generalised Anxiety Disorder (GAD) often struggle with planning and decision-making^110^, and music containing micro-variations, may aid in stepwise planning by facilitating the mental simulation of future events^111^. Furthermore, in some cognitive behavioural therapies such as imagery exposure therapy^35^, the therapist relies on carefully calibrating the vividness of imagined content, and adding or removing micro-variations to accompanying music could help in such scenarios. Outside the clinical domain, professional and recreational activities commonly rely on spatial imagery, such as strategy-based games (e.g., chess^112^) or high-performance sports (e.g., figure skating^113^). Precisely aligning players’ desired levels of mental imagery characteristics through manipulations of background music (e.g., micro-variations) and considering individual traits (e.g., absorption) could further optimise the efficacy of mental imagery techniques and deepen engagement in activities like role-playing^114^. Nonetheless, these applications remain speculative and should be tested through future research, including dual-task paradigms that use more structured and goal-oriented imagery tasks such as role-playing.

Mental imagery also plays a critical role in the aesthetic implications and cognitive processing of music. For composers and producers aiming to create music that fosters vivid imagery, incorporating micro-variations, such as those enabled by the “humanise” control in digital audio workstations, can be a valuable tool. For instance, in genres where creating imagery simulations and immersion is the key, such as film scoring^115^, micro-variations could be strategically used to enhance the narrative or atmospheric qualities of a piece. Similarly, sound engineers and mixers could apply these findings to fine-tune the perceptual qualities of recorded performances, enhancing the human-like qualities of electronic or AI-generated music. In this way, music that engages vivid mental imagery could open new creative possibilities for artists, producers, and engineers.

The musical stimuli used here were deliberately simplistic to control and isolate the constructs of interests. To explore simpler underlying structures, future studies could model micro-variation parameters (micro-timing, strike velocity, and drum-hit location) as continuous predictors to better understand their individual contributions to mental imagery. Additionally, incorporating white noise and random, irregular drumbeats as control conditions could help isolate the effects of these micro-variations. Indeed, while synthesised stimuli here allowed for precise control over parameters, future studies could incorporate performances by expert drummers to enhance ecological validity more and explore how naturally performed quasi-isochronous rhythms compare to algorithmically controlled ones (e.g^93^.,). Moreover, both micro-variation and mechanical conditions were based on acoustic drum simulations, potentially resulting in the micro-variation stimuli sounding more natural. Future work could test this further using ecologically valid yet mechanically timed styles such as electronic dance music.

To increase the generalisability and applied value of the present findings, future studies could incrementally increase the complexity and sophistication of the stimuli and explore other musical features in a controlled environment. For example, future work could build on the current framework by incorporating layered drum patterns, such as those with overlapping rhythmic textures, polyphonic rhythms—where multiple rhythms interact simultaneously—or manipulated harmonic content, to explore how structural, timbral, and rhythmic complexity shape listeners’ mental imagery.

Besides, while the current visual imagery task was intentionally neutral to isolate the effects of micro-variation on imagery, further studies could also explore how emotionally charged tasks (both musically and visually) interact with musical expressivity. Micro-variation potentially could further enhance vividness in emotionally engaging contexts.

## Conclusions

The present study is among the first to systematically explore the effects of specific musical features, here random micro-variation, on the vividness, sentiment, distance and time travelled of the imagined content in a directed mental imagery task. Our findings underscore the ability of music to influence concurrent directed mental imagery, whilst also revealing how individual differences in tendency for absorption, imagining vividly, and musical training interact with such micro-variations in auditory stimuli to shape directed mental imagery characteristics. In addition, the present results further highlight that random micro-variations can greatly enrich music and its impact on listeners, offering important avenues to pursue in therapeutic, creative, and recreational settings.

## Methods

### Participants

One hundred participants (43 females, 55 males, 2 unreported; *M*_*Age*_ = 30.53, *SD*_*Age*_ = 9.21, *Range* = 18 to 65 years) completed the experiment through the online platform Prolific Academic. The sample size was determined based on previous studies employing the same paradigm^14,44,46^. The inclusion criteria were that participants needed to be fluent in English and have normal or corrected-to-normal hearing, and that all participants provided informed consent. Participants’ country of residence was geographically diverse and distributed as follows: Americas (45%) [North America (32%), South America (13%)], Europe (29%), Africa (16%), Oceania (8%), and Asia (2%). Musical expertise of the participants was varied with *M* = 19.1, *SD* = 9.27, *Range* = 7 to 43, calculated through 7 items of the Goldsmiths Musical Sophistication Index’s (Gold-MSI) musical training subscale^90^. Each participant received a reimbursement of 10 GBP. The study adhered to the declaration of Helsinki at all times and received ethics approval from the Western Sydney University Human Research Ethics Committee (H15657).

### Musical stimuli

We recreated the stimulus for this experiment using a recording that was also used in prior studies^77–81^. The original stimulus is a soundtrack titled “15-min solo drumming journey with callback” from Michael Harner’s *Shamanic Journey: Solo and Double Drumming*^116^. It consists of a fast quasi-isochronous naturalistic drumming, with 240 BPM or 4 Hz^77,79^. The original stimulus, performed by humans, contains subtle micro-variations in timing, drum-strike velocity, and drum-hit location. To replicate these characteristics, we recreated all drumming stimuli using Ableton Live 11 Suite^117^, and Chromaphone 2—a synthesiser specially built to have distinct timbres—which includes full physical models of the drums and an option to synthesise performances with random micro-variation^84^.

Recreating the stimulus in a Digital-Audio-Work station allowed for the manipulation and control of the instrumentation of the stimulus, whilst avoiding additional variability (e.g., audio processing artifacts and background noise) that natural recordings would entail^118^. In this way, the internal validity and reliability of our findings would be enhanced while providing a controlled environment to accurately assess the effects of quasi-isochronous rhythms with micro-variation versus mechanical version.

Three different drums were used: a round-frame drum, Chromaphone pre-designed “African Drum” (sound designer: Philippe Derogis), and “Snare Natural” (sound designer: Philippe Derogis). The round-frame drum, 16-inch single-headed drum commonly used in practices of Core Shamanism—a modern framework by Michael Harner that emphasises universal shamanic practices like drumming to induce altered states of consciousness and facilitate mental imagery^77–80,119^—was included for its significance in these practices and its demonstrated effectiveness in this regard. We took a Chromaphone sample instrument, “Arabian Drum” (sound designer: Christian Laffitte), to design the round-frame drum from scratch and emulate the drum sound in the original soundtrack as closely as possible^116^. The adjustments involved modifying the settings for mallet stiffness, noise density, and resonator properties. Additional resynthesis settings in Ableton included Out Hi 127 [to adjust the high-frequency output level to enhance the clarity and presence of the higher frequencies in the sound], Dynamic Tube (84.1% wet signal, -295% envelope, 47.1 ms attack, 23.0 ms release, 9.05 dB drive, -1.90 output level) for the purpose of adding warmth and slight distortion to the sound, and specific Equalisation (EQ) settings (Frequency 4.26 kHz, 0.61 Q factor) to fine-tune its frequency characteristics. Furthermore, in Ableton and for all drums, Dark Snare Room with chambers and large room settings at 30% dry was applied across both random micro-variation and mechanical conditions, to make the audio sound fuller and richer and give the impression that it was recorded with the natural acoustics of a large room.

**Fig. 6:**
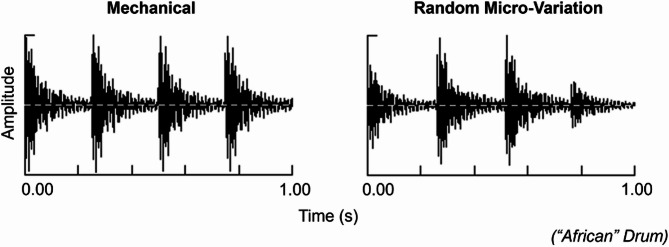
Waveforms of 1-s Mechanical (on the left column) and Random Micro-Variation (on the right column) of quasi-isochronous rhythmic stimuli (240 BPM) played by the “African” drum. The y-axis shows the amplitude, and the x-axis shows the time in seconds. This figure highlights the differences in timing and amplitude patterns between mechanical and random micro-variation conditions, illustrating the subtle variations introduced by the micro-variation condition compared to the uniformity of the mechanical condition.

Each of these drums had both random micro-variation and mechanical versions (see Fig. 6 for example waveforms). Mechanical versions were included to explore the effect of random micro-variations by serving as an unnaturalistic style of the drum patterns and eliminating variation to highlight the specific effects introduced by micro-variation. Additionally, a silence condition as a control was included to distinguish the effects of drum patterns from those specific to the random micro-variation version. The silence version also enabled comparisons with prior studies using the same paradigm but different musical stimuli^14,44^. Several elements were randomised in the micro-variation drum stimuli, including strike velocity, drum-hit location, and timing, to ensure a more naturalistic, humanised variation in the drumming patterns. Three different versions of random micro-variation stimuli were created, with each participant randomly assigned to hear only one version. These micro-variations were drawn from pre-specified random distributions that were piloted to sound natural. Each participant and instrument combination received a unique random draw from these distributions. The following are the parameters of the main controls (i.e., micro-timing, strike velocity, and drum-hit location) as encoded in Ableton and Chromaphone. These parameters differ systematically between the micro-variation and mechanical conditions and serve as the basis for quantifiable contrasts. We either report the actual distribution or the percentage of variability in it. For timing, we used normal (0, 4 ms). In terms of strike velocity, we used the “Velocity” feature on Ableton to add or subtract a random number between 0 and 26 from the original MIDI velocity of each strike. For drum-hit location, we used the “Hit Position” parameter on Chromaphone to modulate the drum-hit location, carefully tuning the drums by hand and ear. This parameter determines the excitation point on the resonator as a percentage of the object’s size. Minimum values correspond to excitation at the object’s border, while maximum values correspond to excitation at its centre. The specified values (frame drum velocity = 19.90%; “African” drum velocity = -60.00%; and snare drum velocity = 15.00%) control how much the velocity influences the hit position, with higher values creating more variation in excitation points. The “Key” (pitch signal) and “Random Signal” (random modulation) controls were both set to 0.00%. Each drum’s hit position in Chromaphone is modulated by the incoming MIDI note’s velocity, which is randomised by Ableton and influenced by the Velocity sub-parameter of Hit Position setting. We also used Chromaphone’s built-in controls, such as mallet stiffness, to modulate loudness and intensity of the drum sound, ensuring natural-sounding results. These modulations were part of the original presets and were not specifically modified.

Regarding the mechanical stimuli, all main randomisation parameters (i.e., strike velocity, drum-hit location, and timing) were set to 0 while keeping other settings the same.

Overall stimuli loudness was normalised to the common value of -23 ± 5*10^–7^ LUFS, as per EBU R-128, using pyloudnorm Python library^120^. The duration of each stimulus was 90 s without a gong at the end. All musical stimuli and the Ableton project are available on OSF, https://osf.io/2qr4n/?view_only=5738a93396434f72bd54736bc801dbb2.

### Procedure and task

The experiment employed a directed mental imagery task, which has been used in prior studies^14,44,46^. The experiment was created in PsychoPy^121^, and the experiment was accommodated on the online platform Pavlovia (www.pavlovia.org), which was then linked to Prolific Academic for participant recruitment.

**Fig. 7:**
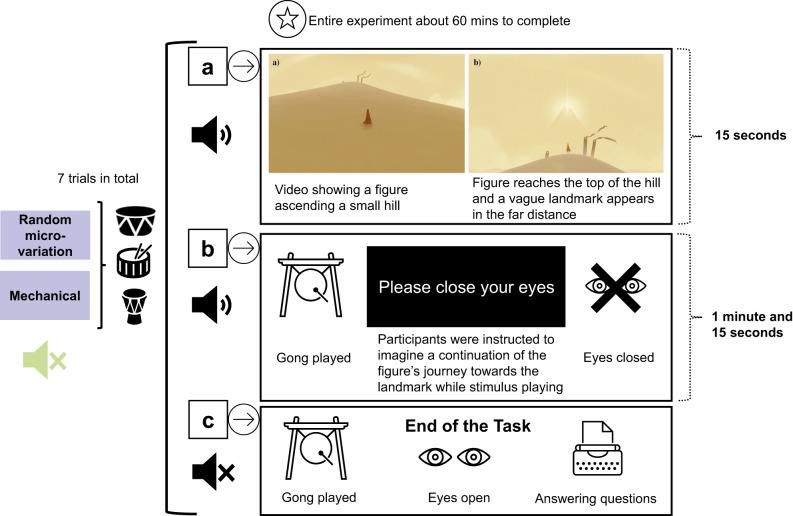
Imagination task. Reproduced with permission from Herff et al.^14^ and Taruffi et al.^46^ Participants were presented with a visual inducer acquired from the video game “Journey” with written permission of Jenova Chen, CEO of ThatGameCompany (https://thatgamecompany.com). The whole experiment took between 30 and 60 min to complete. (**a**) 15-s video illustrates a figure ascending a small hill. Once the figure reaches the top of the hill, a vague landmark appears in the far distance. (**b**) Participants hear a gong-sound and are instructed to close their eyes and imagine a continuation of the figure’s journey towards the landmark. The imagination task lasts one minute and thirty seconds (excluding the initial visual inducer), and is accompanied by a black screen with white lettering stating, “Please close your eyes”. During each trial, participants either listen to quasi-isochronous repetitive drumming with random-micro variation, quasi-isochronous repetitive drumming with a mechanical version, or a silent control condition. (**c**) At the end of the task, the gong-sound is played again, signalling participants to open their eyes and answer a series of questions on time and distance of imagined travel, vividness, sentiment, and content of thoughts.

Participants were presented with a visual inducer consisting of a figure travelling towards a hardly perceptible small hill, with an unclear landmark appearing from the distance once the figure reaches the top of the hill (Fig. 7a). This visual inducer was taken from a video game, “Journey” (https://thatgamecompany.com/), acquired with the permission of *ThatGameCompany.* After 15 s of watching the video, participants were then presented with a gong sound and instructed to close their eyes to imagine a continuation of the figure’s journey in the direction of the landmark (Fig. 7b). This session was accompanied by a black screen with white lettering stating, “Please close your eyes”. During each trial, participants listened to one of six stimuli detailed in the Musical Stimuli section or the silence control condition. Each participant completed a total of seven trials, with the order of conditions randomised. The gong was played at the end of each trial after one minute and thirty seconds, which prompted participants to open their eyes and report on their use of mental imagery through several questions concerning the imagined travel (Fig. 7c):

1. How much time really passed between the two gong sounds? (minutes, seconds)
2. How far away do you estimate the mountain to be at the beginning of the journey? (kilometres, meters)
3. How much time passed in your imagination? (years, months, days, hours, minutes, seconds)
4. How far did you travel in your imagination? (kilometres, meters)
5. How vivid (clear) was the imagery you experienced compared to experiences in real life? (Please indicate a number from 0 = not very clear to 100 = very clear)
6. Please describe your imagination in as much detail as possible. [A free-format text box]

While participants were asked to freely describe their imagined experience for each trial, the task did not constrain them to any specific imagery modality (e.g., visual, kinaesthetic).

At the end of the seven trials, participants were required to complete several self-report questionnaires to assess trait level individual differences in the following order: level of imagery absorption, vividness of visual imagery, and musical training.

The Tellegen Absorption Scale (TAS)^88^ was used to assess the trait level absorption of participants, i.e., the inclination to deeply engage in imaginative or sensory experiences^122^. We focused on the prone to imaginative and altered states subscale due to its strong alignment with our research questions, and its specificity in capturing the dimensions of absorption pertinent to our task. This subscale contains 18 items on a 5-point rating scale, and shows adequate test–retest reliability and internal consistency^96^.

The Vividness of Visual Imagery Questionnaire (VVIQ)^89^ was employed to assess individual variations in the ability to generate and experience vivid visual mental imagery. The questionnaire has 16 items on a 5-point rating scale, assessing the extent to which individuals can mentally visualise and perceive visual images with clarity, detail, and realism^89^. Many studies have shown that VVIQ has a high reliability and validity (e.g^123^.,).

Level of musical training was established using the Musical Training subscale of the Goldsmiths Musical Sophistication Index (Gold-MSI)^90^, which contains seven self-report questions on a 7-point rating scale. The scale demonstrates high test–retest reliability and good internal reliability, mostly through English-speaking participants. It also exhibits reliable correlations with various objective listening ability tests (e.g^124^.,).

The short-form of the Depression Anxiety Stress Scale (DASS-21)^125^ was additionally utilised to explore the influence of mood symptoms on mental imagery. The DASS-21 has 21 items, and demonstrates good reliability and validity in non-clinical adult populations^125^. The DASS results were excluded from the analyses as they belong to a broader study and will be presented in a separate report.

#### Catch trial

At the end of the experiment, a clock-drawing catch trial was included to assess participants’ attentiveness and detect artificial intelligence or automated tools (e.g., “bots”). In this trial, participants were presented with an instruction: “Please use the white box on the right and your mouse to draw a clock face with numbers and the hands at ten past five.” Responses were evaluated based on accuracy, considering the correct placement of the clock hands and the inclusion of necessary numbers for successful completion. In a few instances, participants reversed the hands, placing the minute hand pointing near the five and the hour hand at the two. After carefully checking their overall responses, we accepted these as valid, recognising them as genuine human errors rather than indicators of inattentiveness or automated output.

The whole experiment took around one hour to complete.

### Statistical approach

Data analysis was carried out using Bayesian multilevel regression model to enable modelling each response for the variables (vividness, sentiment, time and distance travelled) while controlling for crossed random effects, such as trial numbers and participants^126^. The model included fixed effects for the interaction between (1) music and silence, (2) random micro-variation, mechanical, and silence, and (3) scores for TAS, VVIQ, and GMSI. Importantly, Sets (1) and (2) were not treated independently. Rather, this structure reflects a hierarchical nesting, where the model first distinguishes between music and silence in (1). Then, conditional on the trial being music, it further models differences between random micro-variation and mechanical conditions in (2). The inclusion of “silence” in (2) is a modelling strategy to support faster convergence and does not imply independent treatment. The random effects controlled for trial numbers, participants, and the nested interactions among (1), (2), (3) instruments (including frame, snare, or “African”) or silence, and (4) which micro-variation (1, 2, or 3) was taken into account. While instrument type was not a primary focus of the current study, analyses exploring its role are reported in the supplementary materials (available via OSF, https://osf.io/2qr4n/?view_only=5738a93396434f72bd54736bc801dbb2). These analyses follow the statistical approach employed in previous studies using the same paradigm^14,44,46^. All analyses were implemented in R^127^, using the brms package^128^.

Following previous auditory perception work^14,44^, continuous variables were standardised (*M* = 0, *SD* = 1), and the imagined time and distance travelled responses by the participants were natural log-scaled to be standardised. A weakly informative prior (a Student’s t-distribution with a mean of 0, a standard deviation of 1, and 3 degrees of freedom) was set, and the models ran with 4,000 warm-ups, and 10,000 iterations on 4 Markov chains. All R-hats were 1.00, indicating full convergence. Here, hypotheses are reported with the model coefficients (*β*) related to certain hypotheses, the estimated error for these coefficients (*EE*_*β*_), the evidence ratio in favour of that certain hypothesis (*Odds*_*β*_), and their Posterior Probability (*Post.Prob*). Average predictions were generated and evaluated to draw inference^129^. For convenience, we mark effects deemed strong evidence at a 5% alpha level with * (i.e., evidence ratio ≥ 19 (*Post.Prob* ≥ 0.95) for one-sided or ≥ 39 (*Post.Prob* ≥ 0.98) for two-sided tests; see^85,91^). For hypotheses with a clear directional expectation (e.g., random micro-variation > mechanical > silence or high > low scale test scores, we ran one-sided tests (*Odds* ≥ 19). For analyses without a clear directional expectation (e.g., interactions between conditions and scales), we applied a more stringent threshold (*Odds* ≥ 39) for significance and ran two-sided tests.

Imagined sentiment from the free-format responses was first extracted by Natural Language Toolkit (NLTK)^86^ and the Valence Aware Dictionary for sEntiment Reasoning (VADER) model^87^ in Python3. The VADER provides an output of emotional valence and intensity in a continuum from negative to positive through mapping lexical features, higher numerical values indicating a more positive sentiment. VADER is trained on a pre-defined lexicon of around 7,500 words and phrases, applying rule-based heuristics for a fast sentiment analysis.

## Data Availability

All data, analytical scripts, the Ableton project, and musical stimuli are available on OSF, https://osf.io/2qr4n/?view_only=5738a93396434f72bd54736bc801dbb2.
